# T1DiabetesGranada: a longitudinal multi-modal dataset of type 1 diabetes mellitus

**DOI:** 10.1038/s41597-023-02737-4

**Published:** 2023-12-20

**Authors:** Ciro Rodriguez-Leon, Maria Dolores Aviles-Perez, Oresti Banos, Miguel Quesada-Charneco, Pablo J. Lopez-Ibarra Lozano, Claudia Villalonga, Manuel Munoz-Torres

**Affiliations:** 1 University of Granada, Research Center for Information and Communication Technologies, Granada, 18014 Spain; 2 University of Cienfuegos, Department of Computer Science, Cienfuegos, 55100 Cuba; 3 University Hospital Clínico San Cecilio, Endocrinology and Nutrition Unit, 18016 Granada, Spain; 4 Instituto de Salud Carlos III, CIBER on Frailty and Healthy Aging (CIBERFES), 28029 Madrid, Spain; 5 Instituto de Investigación Biosanitaria de Granada (ibs.GRANADA), 18014 Granada, Spain; 6 University of Granada, Department of Medicine, Granada, 18016 Spain

**Keywords:** Type 1 diabetes, Health care

## Abstract

Type 1 diabetes mellitus (T1D) patients face daily difficulties in keeping their blood glucose levels within appropriate ranges. Several techniques and devices, such as flash glucose meters, have been developed to help T1D patients improve their quality of life. Most recently, the data collected via these devices is being used to train advanced artificial intelligence models to characterize the evolution of the disease and support its management. Data scarcity is the main challenge for generating these models, as most works use private or artificially generated datasets. For this reason, this work presents T1DiabetesGranada, an open under specific permission longitudinal dataset that not only provides continuous glucose levels, but also patient demographic and clinical information. The dataset includes 257 780 days of measurements spanning four years from 736 T1D patients from the province of Granada, Spain. This dataset advances beyond the state of the art as one the longest and largest open datasets of continuous glucose measurements, thus boosting the development of new artificial intelligence models for glucose level characterization and prediction.

## Background & Summary

Diabetes mellitus (DM) is a metabolic and chronic disease characterized by chronic hyperglycemia. There are mainly two types of DM: type 1 diabetes mellitus (T1D) and type 2 diabetes mellitus (T2D). One of the main differences between these two types are the age of onset and the treatment. T1D usually occurs in younger people than T2D, although in recent years there has been an increase in cases of T1D in adults. Patients with T1D have to be treated with insulin but this is almost never the case for patients with T2D^1,2^. In general, having control of the blood glucose level (BGL) is easier for T2D patients than for T1D patients. To control their BGLs, T1D patients must keep strict control of the amount of carbohydrates ingested, physical activity performed, and insulin administered, which can become very complex^3,4^.

In the light of these challenges, scientific and technological efforts have been recently made to improve the quality of life of people with DM. Most notorious examples are the development of wearable devices such as insulin pumps, continuous glucose meters (CGM), and flash glucose meters (FGM). In addition, mobile or wearable devices, such as wristbands or chest straps, are also used to measure other important variables for the disease like physical activity^5^. In fact, the use of CGM and FGM has led to a significant improvement in controlling BGLs in T1D patients^6^. The use of these devices by T1D patients is beneficial as it provides objective and continuous data that can help doctors to treat more effectively, but also offers the opportunity to use artificial intelligence and data science techniques to reveal interesting patterns from this data. In this regard, the most relevant applications would be to accurately predict patients’ BGLs in the short and mid term and to forecast the occurrence of hypoglycemia and hyperglycemia in advance. Comprehensive longitudinal datasets become essential to support this type of applications.

Despite the advent in the use of CGM and FGM in the recent years, there is a clear lack of open, longitudinal datasets presenting data collected by these devices. A great deal of research in this area builds on private datasets, some of which obtain the data from real CGM or FGM sensors^7–13^, and others generate the data artificially (*in silico*)^14–18^. Few datasets are found in the literature that meet some of the necessary requirements for making realistic predictions: “REPLACE-BG”^19^, a public dataset collecting real CGM data during 182 days from 226 T1D patients with well-controlled DM; “The D1NAMO Open Dataset”^20^, an open dataset collecting real CGM data during approximately 30 days from 9 T1D patients; “The OhioT1DM Dataset”^21^, an on-request dataset collecting real CGM data during 56 days from 12 T1D patients; and “ShanghaiT1DM”^22^ a public dataset collecting real CGM data during 14 days from 12 T1D patients. However, these four datasets are characterized by a relatively small sample size and short study duration. Very recently, a contribution has been made towards increasing the study duration: “DiaTrend”^23^, a public dataset collecting real CGM data during an average of 510 days from 54 T1D patients.

In view of the scarcity of open datasets in this domain and the limitations of existing ones, we contribute, to the best of our knowledge, with the longest and largest open under specific permission longitudinal dataset of FGM sensor data. The dataset comprises 257 780 days of FGM sensor measurements collected over four years from 736 T1D patients, providing also patient demographic and clinical information. The collected data spans several years and can therefore be used to investigate the disease evolution of T1D patients at different times of the year, for example, to make comparisons between holidays and regular days, or between climatic seasons. Moreover, this dataset is made available to the scientific community to boost the development of new artificial intelligence models. For example, it can be used to automatically determine patient profiles to provide more personalized treatment, or to predict the disease evolution to implement anticipatory and preventive diabetes management strategies.

## Methods

### Ethical approval

This study was reviewed and approved by the Ethics Committee of Biomedical Research of the Province of Granada (CEIm/CEI GRANADA). Protocol code: K134665CRL, Ethics portal code: 0698-N-21. The data has been approved to be published under a Data Usage Agreement.

### Participant onboarding

Participants had to be patients at the Clinical Unit of Endocrinology and Nutrition of the San Cecilio University Hospital of Granada, Spain. They had to be patients with T1D and be selected to wear a *FreeStyle Libre* device (*Abbott Diabetes Care, Inc., Alameda, CA, USA*). The study began on January 6th, 2018, and ended on March 21st, 2022, eventually involving 736 patients.

The enrolment into the study began when the patient was informed that had been selected to use the FGM, because they met the required eligibility criteria. Then, the patient visited the Clinical Unit of Endocrinology and Nutrition and received an explanation of how the sensor worked and, its potential for T1D management and the handling of the collected data. During this visit, patients were asked to give their consent to participate in the study and were informed of the possibility that their data would be shared anonymously. This was followed by a training session where the patient learnt how to wear and, operate the device and upload the data to the cloud platform. To upload the data, the patient had to register on the system and give consent for their BGL measurements and demographic information to be used for research purposes. They consented to access, use, and share the anonymized data. Should a participant wanted to withdraw from the study they had to unregister from the system or make a specific request. From then on, no further data was collected. However, the anonymized collected data was persisted. In any case, no participant withdrew from the study.

### Data collection

The most commonly used FGM device during the study was *FreeStyle Libre 2*, although its first version, *FreeStyle Libre*, was also used in some cases at the beginning of the study. Both versions of the device are very similar and are manufactured by *Abbott Diabetes Care, Inc., Alameda, CA, USA*^24^. These devices have a sensor in the form of a tiny needle that when introduced into the tissue measures the glucose level in the interstitial fluid. Each device has a service life of 14 days, during which it is not necessary to recharge the battery or perform any other action on it. After these 14 days, the device must be replaced with a new one.

Measurements of glucose in the interstitial fluid are recorded at 15-minute intervals. These measurements are stored in the device memory, which can hold a maximum of 8 hours of data. Before these 8 hours have elapsed, it is necessary to scan the FGM with a Near Field Communication (NFC) device, either a mobile phone or a *FreeStyle Libre Reader*. Once the scan takes place, the data is copied from the FGM device to the NFC device. Also, each time a patient performs an NFC scan, the current BGL value is added to the data as an extra measurement point. Then, when the NFC device used to collect the measurements is connected to the Internet, the data is transferred to the *LibreView* cloud platform.

In addition to BGLs, demographic and clinical information from patients were also collected in the study. The first time a patient visits the Clinical Unit of Endocrinology and Nutrition, data, such as birth date, pathological history of other diseases, home address and contact telephone numbers, are collected. For privacy reasons and relevance to T1D, only year of birth and additional diagnoses of other diseases were included in the dataset. The physicians of the endocrinology unit schedule the following visits to the clinic considering the status of each patient (e.g. every three months or every six months) and request a series of biochemical tests for the days prior to the consultation to measure their biochemical parameters. The values of the patients’ biochemical parameters obtained during the study period were also included as part of the dataset.

### Data preparation

Independent technicians from the Information Technology Service of the San Cecilio University Hospital of Granada were designated for conducting the data anonymization process. They eliminated information that was confidential to the patients and irrelevant to the study such as name, e-mail, and medical record numbers. In addition, they assigned each patient a unique identifier to avoid revealing the identity of the patient.

Some basic data cleaning tasks were performed on the anonymized data, such as removal of duplicate rows, removal of rows with relevant missing values, and removal of irrelevant or empty columns. Furthermore, column names were translated from Spanish (local language) to English and some variables values were recoded into English, for example sex and the names of the biochemical parameters. Patients’ diagnoses had an associated code and description following the standard of the Spanish government’s Ministry of Health^25^, so a mapping of the codes to the equivalent English version of the standard^26^ was performed. In addition, the values of some variables were reformatted. For instance the date fields in DD-MM-YYYY format were transformed to YYYYY-MM-DD format to optimize tasks like sorting. Finally, all the files that compose the dataset were sorted by patient identifier and date if available.

## Data Records

The dataset is available for open access under specific permission via the Zenodo repository T1DiabetesGranada: a longitudinal multi-modal dataset of type 1 diabetes mellitus^27^. The data is stored in four comma-separated values (*CSV*) files which are presented in Table 1 and described in detail below.

**Table 1:** Overview of the T1DiabetesGranada dataset.

File Name	Number of records	Number of variables	Size (Bytes)	Lost values	Description
Patient_info.csv	736	11	8 096	Yes	File containing information about the patients.
Glucose_measurements.csv	22 671 708	4	90 686 832	No	File containing the continuous blood glucose level measurements of the patients.
Biochemical_parameters.csv	87 482	4	349 928	No	File containing data of the biochemical tests performed on patients to measure their biochemical parameters.
Diagnostics.csv	1 757	3	5 271	No	File containing diagnoses of diabetes mellitus complications or other diseases that patients have in addition to type 1 diabetes mellitus.

### Patient information

**Fig. 1:**
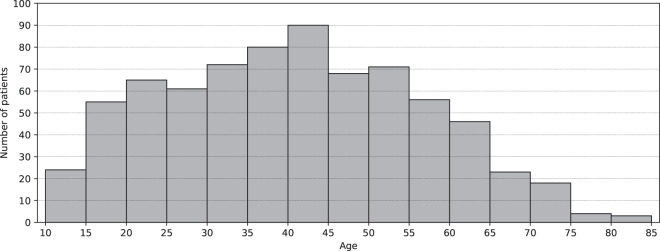
Distribution of the age of the patients at the start of the data collection (January 6th, 2018).

**Fig. 2:**
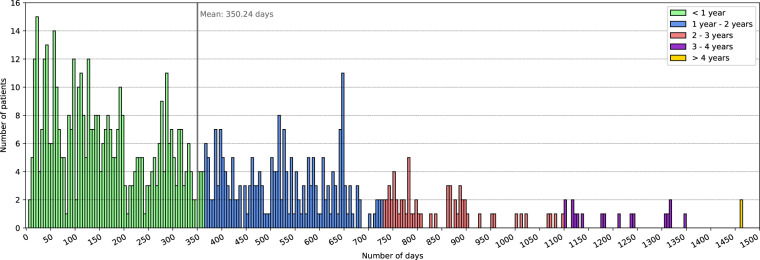
Number of patients by number of days with blood glucose level measurements. The colors depict periods of one year. The mean number of days is represented by a vertical line.

**Fig. 3:**
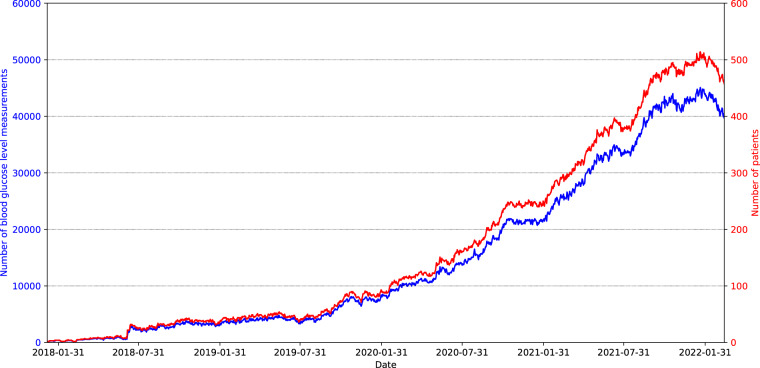
Number of patients and blood glucose level measurements by date.

*Patient_info.csv* is the file containing information about the patients, such as demographic data, start and end dates of BGL measurements and biochemical parameters, number of biochemical parameters or number of diagnostics. This file is composed of 736 records, one for each patient in the dataset. Table 2 shows the detail of the eleven variables that make up the file *Patient_info.csv*. “Patient_ID” is an alphanumeric variable that uniquely identifies the patients in all files of the dataset. “Sex” codifies the sex of the patient and the distribution of this variable is balanced in the sample with 373 female patients (50.68%) and 363 male patients (49.32%). “Birth_year” indicates the year of birth of the patient and ranges from 1936 to 2005. The age of the patients at the beginning of the study (January 6th, 2018) was 40.34 ± 15.77 years and ranged from 12 to 81 years. The distribution of the patients’ age is represented in Fig. 1. “Initial_measurement_date” and “Final_measurement_date” mark the date of the first and the last BGL measurement of each patient in the study. This information is extracted from the file *Glucose_measurements.csv* by searching for the date of the earliest and the latest BGL measurement of each patient. “Number_of_days_with_measures” is the number of days with measurements per patient, this means the number of days in which the patient has at least one BGL measurement, with an average of 350.24 ± 284.15 days. This information is extracted from the file *Glucose_measurements.csv* and the histogram of this variable is shown in Fig. 2. “Number_of_ measurements” represents the total number of BGL measurements per patient, with an average of 30802.95 ± 25704.87, and the information was extracted from the file *Glucose_measurements.csv*. Figure 3 depicts the number of patients participating in the study and the number of BGL measurements collected across time. Both the number of patients and the number of measurements have increased since the beginning of the study. “Initial_biochemical_parameters_date” and “Final_biochemical_parameters_date” are the dates of the first and the last time a biochemical parameter is measured for each patient. This information is extracted from the file *Biochemical_parameters.csv* by searching for the date of the earliest and the latest value of biochemical parameter of each patient. “Number_of_biochemical_parameters” represents the number of biochemical parameters values per patient. The average of this variable is 120.00 ± 87.83 calculated over the 723 patients that have available some values. This information is extracted from the file *Biochemical_parameters.csv*. “Number_of_diagnostics” represents the number of diagnostics per patient. The average of this variable is 3.44 ± 2.95 calculated over the 511 patients that have available some diagnostics. This information is extracted from the file *Diagnostics.csv*.

**Table 2:** Variables detail from *Patient_info.csv* file.

Variable name	Type	Values	Description
Patient_ID	String	LIB19XXXX	Unique identifier of the patient.
Sex	String	One of {‘F’, ‘M’}	Sex of the patient.
Birth_year	Date	YYYY	Year of birth of the patient.
Initial_measurement_date	Date	YYYY-MM-DD	Date of the first blood glucose level measurement of the patient in the *Glucose_measurements.csv* file.
Final_measurement_date	Date	YYYY-MM-DD	Date of the last blood glucose level measurement of the patient in the *Glucose_measurements.csv* file.
Number_of_days_with_measures	Integer	8 to 1 463	Number of days with blood glucose level measurements of the patient, extracted from the *Glucose_measurements.csv* file.
Number_of_measurements	Integer	400 to 137 292	Number of blood glucose level measurements of the patient, extracted from the *Glucose_measurements.csv* file.
Initial_biochemical_parameters_date	Date	YYYY-MM-DD	Date of the first biochemical test to measure some biochemical parameter of the patient, extracted from the *Biochemical_parameters.csv* file.
Final_biochemical_parameters_date	Date	YYYY-MM-DD	Date of the last biochemical test to measure some biochemical parameter of the patient, extracted from the *Biochemical_parameters.csv* file.
Number_of_biochemical_parameters	Integer	4 to 846	Number of biochemical parameters measured on the patient, extracted from the *Biochemical_parameters.csv* file.
Number_of_diagnostics	Integer	1 to 24	Number of diagnoses realized to the patient, extracted from the *Diagnostics.csv* file.

### Glucose measurements

**Fig. 4:**
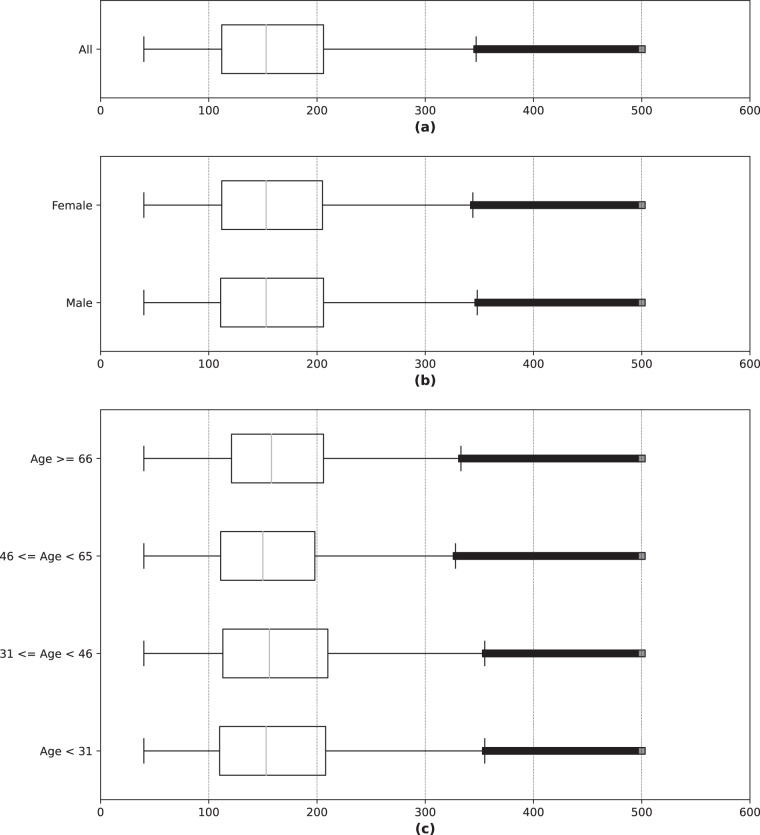
Data distribution of the blood glucose level measurements: (**a**) Overall; (**b**) By sex; and (**c**) By age.

**Fig. 5:**
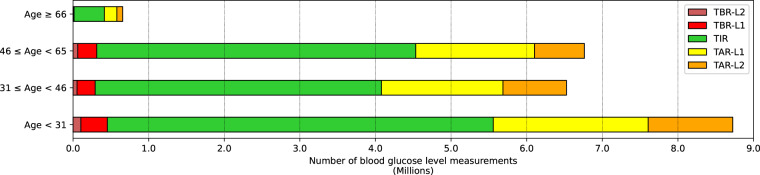
Percentage of blood glucose level measurements per level ranges and age ranges. Blood glucose level ranges are represented by colors and defined as: TBR-L2, time below range with level 2 hypoglycemia (<54 mg/dL); TBR-L1, time below range with level 1 hypoglycemia (54 mg/dL - 69 mg/dL); TIR, time in range (70 mg/dL - 180 mg/dL); TAR-L1, time above range with level 1 hyperglycemia (181 mg/dL - 250 mg/dL). TAR-L2: time above range with level 2 hyperglycemia (≥251 mg/dL).

*Glucose_measurements.csv* is the file containing the continuous BGL measurements of the patients. The file is composed of more than 22.6 million records that constitute around 257 780 days of continuous BGL measurements. In this file there are multiple records with the same “Patient_ID” since each patient has several BGL measurements, usually one every 15 minutes. Table 3 describes the variables that make up the file *Glucose_measurements.csv*. “Measurement_date” and “Measurement_time” are the date and time in which the measurement of the BGL takes place. “Measurement” is the value of the BGLs of the patient measured in mg/dL and it is observed to be in average 164.78 ± 71.57 mg/dL. Figure 4a illustrates the distribution of the BGL measurements across the entire sample. Most of the values are between 100 mg/dL and 200 mg/dL with a median close to 150 mg/dL, and values above 350 mg/dL are considered extreme. It is also interesting to analyze the distribution of continuous BGL measurements in relation to other variables. Figure 4b depicts the data distribution stratified by sex, while Fig. 4c illustrates the distribution according to various age ranges. Both figures show that the distribution of BGL values exhibits minimal variation with respect to gender and age range, mirroring the overall sample pattern depicted in Fig. 4a. Doctors normally consider some specific BGL ranges: time below range (TBR), time in range (TIR), and time above range (TAR). Standardized metrics for the use of CGM for clinical care compute the percentage of BGL measurements and time as^28^: < 54 mg/dL (TBR, level 2 hypoglycemia); 54–69 mg/dL (TBR, level 1 hypoglycemia); 70–180 mg/dL (TIR); 181–250 mg/dL (TAR, level 1 hyperglycemia); > 250 mg/dL (TAR, level 2 hyperglycemia). Figure 5 displays the count of BGL measurements within specific BGL intervals and age groupings for the sampled population. The majority of the BGL measurements are in TIR, and the out-of-range values are mostly TAR. Finally, as previously noted, Fig. 3 shows the connection between the count of participating patients and the quantity of BGL measurements over time. The blue plot clearly depicts an upward trend, signifying a growth in the daily count of continuous BGL measurements as time progresses. This phenomenon is largely attributable to the concurrent growth in the number of patients, as indicated by the red plot, hence resulting in the increase of the number of daily BGL measurements.

**Table 3:** Variables detail from *Glucose_measurements.csv* file.

Variable name	Type	Values	Description
Patient_ID	String	LIB19XXXX	Unique identifier of the patient.
Measurement_date	Date	YYYY-MM-DD	Date of the blood glucose level measurement.
Measurement_time	Time	HH:MM:SS	Time of the blood glucose level measurement.
Measurement	Integer	40 to 500	Value of the blood glucose level measurement in mg/dL.

### Biochemical parameters

**Fig. 6:**
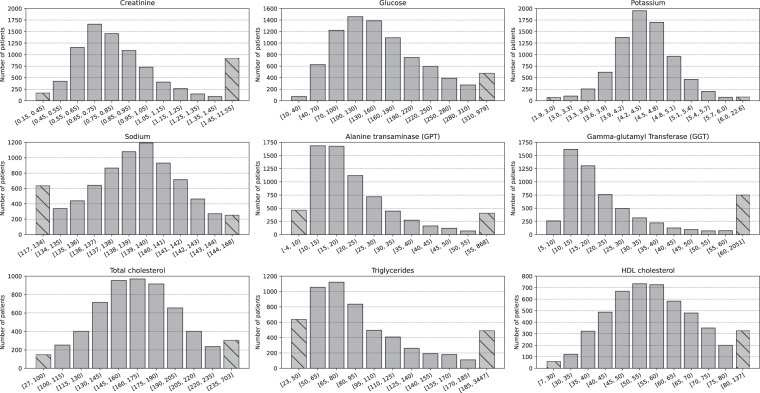
Distribution of the values of the most commonly measured biochemical parameters. The bars with lines do not have the same range breadth as the rest in each chart.

*Biochemical_parameters.csv* is the file containing data of the biochemical tests performed on patients to measure their biochemical parameters. This file is composed of 87 482 records. A patient, identified by their “Patient_ID”, can have more than one record in this file, one for each biochemical parameter measured on the patient throughout the study. Table 4 explains the variables that make up the *Biochemical_parameters.csv* file. “Reception_date” is the date when the sample to measure the biochemical parameter is received in the laboratory. “Name” indicates the name of the measured biochemical parameter and “Value” the value of the biochemical parameter. Throughout the study, 17 different types of biochemical parameters were measured. Table 5 shows the measurement units of these biochemical parameters. Table 6 provides a summary of statistical information regarding the count of biochemical parameters per patient. The most prevalent biochemical parameters are “creatinine” with an average of 11.54 ± 12.05 occurrences and “glucose” with an average of 11.34 ± 11.63 occurrences. Conversely, the least frequently encountered parameters are “IA2 ANTIBODIES” with an average of 0.09 ± 0.30 occurrences and “insulin” with an average of 0.09 ± 0.41 occurrences. Furthermore, Table 7 presents a summary of statistical data pertaining to the values of these biochemical parameters. Figure 6 depicts the distribution in the sample of the values of the nine most common biochemical parameters.

**Table 4:** Variables detail from *Biochemical_parameters.csv* file.

Variable name	Type	Values	Description
Patient_ID	String	LIB19XXXX	Unique identifier of the patient.
Reception_date	String	YYYY-MM-DD	Date of receipt in the laboratory of the sample to measure the biochemical parameter.
Name	String	One of {‘Potassium’, ‘HDL cholesterol’, ‘Gamma-glutamyl Transferase (GGT)’, ‘Creatinine’, ‘Glucose’, ‘Uric acid’, ‘Triglycerides’, ‘Alanine transaminase (GPT)’, ‘Chlorine’, ‘Thyrotropin (TSH)’, ‘Sodium’, ‘Glycated hemoglobin (Ac)’, ‘Total cholesterol’, ‘Albumin (urine)’, ‘Creatinine (urine)’, ‘Insulin’, ‘IA ANTIBODIES’}	Name of the measured biochemical parameter.
Value	Float	−4.0 to 6446.74	Value of the biochemical parameter.

**Table 5:** Measurement unit of the biochemical parameters in *Biochemical_parameters.csv* file.

Biochemical parameter name	Measurement unit
Potassium	mEq/L
HDL cholesterol	mg/dL
Gamma-glutamyl Transferase (GGT)	U/L
Creatinine	mg/dL
Glucose	mg/dL
Uric acid	mg/dL
Triglycerides	mg/dL
Alanine transaminase (GPT)	U/L
Chlorine	mEq/L
Thyrotropin (TSH)	*μ*UI/mL
Sodium	mEq/L
Glycated hemoglobin (Ac)	%
Total cholesterol	mg/dL
Albumin (urine)	mg/dL
Creatinine (urine)	mg/dL
Insulin	*μ*UI/mL
IA ANTIBODIES	U/mL

**Table 6:** Summary of statistics of the number of biochemical parameters per patient.

Biochemical Parameter	Mean	STD	Median	Minimum	Maximum
Alanine transaminase (GPT)	9.69	8.85	8	0	90
Albumin (urine)	5.82	2.88	6	0	21
Chlorine	2.63	6.40	1	0	74
Creatinine	11.54	12.05	9	0	115
Creatinine (urine)	5.99	3.15	6	0	27
Gamma-glutamyl Transferase (GGT)	8.27	7.68	7	0	89
Glucose	11.34	11.63	9	0	111
Glycated hemoglobin (A1c)	6.49	2.76	7	0	16
HDL cholesterol	6.88	3.48	7	0	26
IA2 ANTIBODIES	0.09	0.30	0	0	2
Insulin	0.09	0.41	0	0	6
Potassium	10.67	11.72	8	0	108
Sodium	10.62	11.54	8	0	107
Thyrotropin (TSH)	6.21	3.13	6	0	17
Total cholesterol	8.09	4.92	7	0	40
Triglycerides	7.87	4.65	7	0	36
Uric acid	6.57	4.81	6	0	41

**Table 7:** Summary of statistics of the biochemical parameters values.

Biochemical Parameter	Mean	STD	Median	Min	Max
Alanine transaminase (GPT)	26.98	44.62	19.00	−4.00	868.00
Albumin (urine)	4.86	26.56	0.70	0.01	992.20
Chlorine	102.96	5.34	103.00	76.00	136.00
Creatinine	1.04	0.95	0.80	0.15	11.55
Creatinine (urine)	114.14	66.80	101.41	2.00	543.46
Gamma-glutamyl Transferase (GGT)	43.80	113.55	19.00	5.00	2051.00
Glucose	163.04	85.40	146.00	10.00	979.00
Glycated hemoglobin (A1c)	7.82	1.39	7.60	4.00	18.00
HDL cholesterol	56.63	14.37	55.00	7.00	137.00
IA2 ANTIBODIES	409.64	1237.70	1.15	0.21	6446.74
Insulin	22.69	40.54	10.24	0.74	298.50
Potassium	4.43	0.66	4.40	1.90	22.60
Sodium	138.16	3.47	138.00	117.00	168.00
Thyrotropin (TSH)	2.87	4.68	2.22	0.01	204.89
Total cholesterol	169.45	39.93	167.00	27.00	703.00
Triglycerides	102.97	94.69	81.00	23.00	3447.00
Uric acid	4.39	1.54	4.20	1.00	14.20

**Table 8:** Variables detail from *Diagnostics.csv* file.

Variable name	Type	Values	Description
Patient_ID	String	LIB19XXXX	Unique identifier of the patient.
Code	String	594 unique values	ICD-9-CM diagnosis code.
Description	String	594 unique values	ICD-9-CM long description.

### Diagnostics

**Fig. 7:**
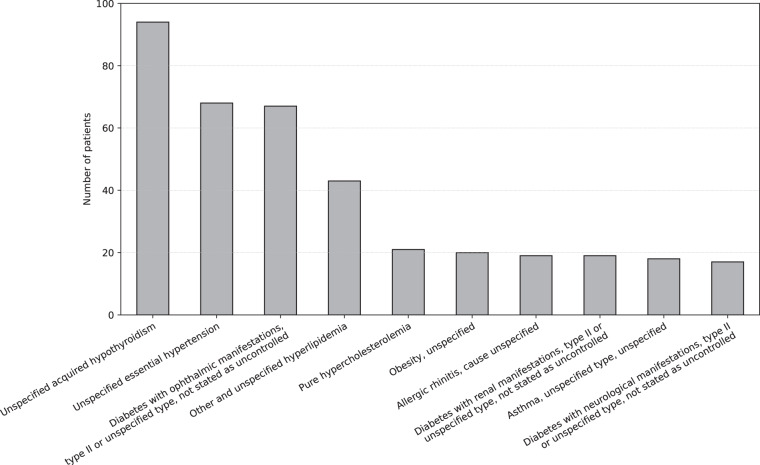
Number of patients per most common diagnoses of diabetes mellitus complications or other diseases.

*Diagnostics.csv* is the file containing diagnoses of DM complications or other diseases that patients have in addition to T1D. This file is composed of 1 757 records. A patient, identified by their “Patient_ID”, can have more than one record in this file, as many as diagnoses. Table 8 describes the variables that make up the file *Diagnosis.csv*. The diagnoses are represented by the ICD-9-CM standard code^26^ in the variable “Code” and the ICD-9-CM long description in “Description”. In the *Diagnostics.csv* file there are 594 different types of diagnoses, Fig. 7 shows the distribution of the ten most common ones.

## Technical Validation

BGL measurements are collected using *FreeStyle Libre* devices, which are widely used for healthcare in patients with T1D. *Abbott Diabetes Care, Inc., Alameda, CA, USA*, the manufacturer company, has conducted validation studies of these devices concluding that the measurements made by their sensors compare to YSI analyzer devices (*Xylem Inc*.), the gold standard, yielding results of 99.9% of the time within zones A and B of the consensus error grid^29^. In addition, other studies external to the company concluded that the accuracy of the measurements is adequate^30^.

Moreover, it was also checked in most cases the BGL measurements per patient were continuous (i.e. a sample at least every 15 minutes) in the *Glucose_measurements.csv* file as they should be.

## Usage Notes

The dataset is open under specific permission for research purposes in the Zenodo repository T1DiabetesGranada: a longitudinal multi-modal dataset of type 1 diabetes mellitus^27^. For data downloading, it is necessary to be authenticated on the Zenodo platform, accept the Data Usage Agreement and send a request specifying full name, email, and the justification of the data use. This request will be processed by the Secretary of the Department of Computer Engineering, Automatics, and Robotics of the University of Granada and access to the dataset will be granted.

The files that compose the dataset are CSV type files delimited by commas and are available in *T1DiabetesGranada.zip*. A Jupyter Notebook (Python v. 3.8) with code that may help to a better understanding of the dataset, with graphics and statistics, is available in *UsageNotes.zip*.

### Limitations

The current dataset faithfully represents the evolution of glucose levels over the time of the study. We firmly believe that these continuous glucose measurements are useful to researchers in the field, however there are some limitations to consider.

**Fig. 8:**
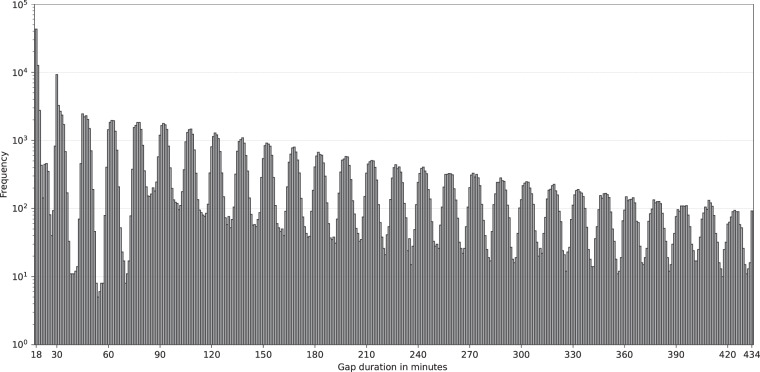
Logarithmic distribution of the gaps of blood glucose level measurements.

During the patient participation in the study there may be data gaps without BGL measurements due to two main reasons. The first reason is when the patient does not scan the FGM with an NFC device in less than 8 hours. Then, the BGL measurements are overwritten in the internal memory, thus losing the oldest data. The second reason is when the patient, after the 14-day life span of the FGM device, does not activate the replacement device early enough. Nonetheless these two situations were already considered in the protocol design in order to ensure that patients would proceed accordingly and do not lose data. Although BGL measurements are normally recorded every 15 minutes, there might be slight variations due to the tolerance of the device (±1 min). Hence, measurement gaps are considered when the intervals are above 17 minutes. These gaps represent the 0.95% of the BGL measurements. Figure 8 shows the frequency of gaps of duration from 18 and 434 minutes as these represent statistically the majority of detected gaps.

The duration of the participation of the patients varies due two main reasons. Firstly, the patients’ enrolment in the study was progressive because the capacity to process all patients with T1D by the Clinical Unit of Endocrinology and Nutrition of the San Cecilio University Hospital of Granada is limited. The enrolment was done in order of priority depending on the health status of the patients. Secondly, in seldom cases, the data collection ended due to different reasons: patients abandoned the use of the FGM device due to allergy to the glue used to attach it to the skin, death of the patient, transfer of the patient to another clinical unit, or the patient’s personal decision to no longer use the FGM device.

## Data Availability

The data described in this manuscript was generated using some custom code located in *CodeAvailability.zip* of the Zenodo repository T1DiabetesGranada: a longitudinal multi-modal dataset of type 1 diabetes mellitus^27^. The code is provided as Jupyter Notebooks created with Python v. 3.8. The code was used to conduct the tasks described in sections Data preparation and Data Records, such as data curation and transformation, and variables extraction.
